# The use of complementary and alternative medicine for functional gastrointestinal disorders among the saudi population

**DOI:** 10.1016/j.jsps.2024.102084

**Published:** 2024-04-24

**Authors:** Salmeen D. Babelghaith, Ibrahim Sales, Wajid Syed, Mohamed N. Al-Arifi

**Affiliations:** Department of Clinical Pharmacy, College of Pharmacy, King Saud University, P.O. Box 2454, Riyadh 11451, Saudi Arabia

**Keywords:** Complementary and alternative medicine, Functional gastrointestinal disorders, Saudi population, Constipation, Ginger

## Abstract

**Background and objective:**

Complementary and alternative medicine (CAM) is a common practice among patients, who experience functional gastrointestinal disorders (FGID). Among the Saudi population, less is known about CAM use for FGID. Therefore, this study aimed to determine the prevalence of CAM utilization for FGID amongst the Saudi population and determine the types of CAM used for treatment.

**Method:**

A cross-sectional study was carried out in Riyadh, Saudi Arabia during February 2023 through social media platforms using questionnaires adopted from the literature. There were three sections in the questionnaire including demographic information, questions to determine the prevalence of CAM use for FGID, the types of FGID, and the types of CAM utilization, and questions on the sources of information about CAM. Multivariable logistic regression was applied to find factors associated with CAM use. All statistical analyses were performed using SPSS version 26.

**Results:**

A total of 828 people participated in this study. The overall prevalence of CAM use for FGID problems was 87.2 %. There were no significant differences in CAM use for FGID problems between men (87.5 %) and women (86.3 %) (P = 0.727). The most commonly used types of CAM for FGID were ginger (73.4 %), chamomile (66.6 %), mint (61.6 %), turmeric (59.0 %), anise (55.5 %), fennel (43.1 %), and Activia yogurt©️ (42.7 %). The most common FGID disorders for utilizing CAM were IBS (29.9 %), followed by constipation (29.8 %), dyspepsia (22.7 %), and bloating (17.0 %). In the multivariable regression, age, gender and employment status did not have an impact on the odds of using CAM. The subjects who had high school, university, and postgraduate education had significant odds ratios of CAM use (OR = 2.73; 95 % CI: 1.22–6.13), (OR = 4.18; 95 % CI: 2.03–8.58), and (OR = 20.85; 95 % CI: 5.51–78.80), respectively, compared to subjects who did not complete high school. Participants who had private insurance had a significant odds ratio (OR = 0.27; 95 % CI: 0.14–0.55) compared to governmental insurance.

**Conclusion:**

The use of CAM among the Saudi population is alarmingly high; however, the lack of standardized medical recommendations and treatment options may be the cause. Although there were no significant gender differences, participants with higher educational levels and private insurance coverage were more likely to use CAM for FGID. Patients suffering from FGID and limited access to medical advice and treatment options are vulnerable to being exposed to dubious and incredible information sources. Expanding access to preventative medical services, funding governmental medical websites to provide credible information, educating healthcare professionals about FGID, and conducting more research in safe and effective treatments for FGID is recommended.

## 1 Introduction

Complementary and alternative medicine (CAM) is a broad category of treatments, procedures, and supplies that claim to prevent or treat disease (Aljawadi et al., 2020). Because there is inadequate evidence to support the safety and efficacy of CAM therapies, they are not a part of standard medical care (Aljawadi et al., 2020, Barnes et al., 2008). Alternative interventions are utilized in place of traditional medicine, while complementary interventions are used in addition to conventional therapy (Barnes, Bloom and Nahin, 2008). The most common types of CAM are herbal products, acupuncture, medical massage, homeopathy, chiropractic treatment, and reflexology (Kemppainen et al., 2018, Frass et al., 2012).

The use of CAM among the general population is widespread globally (Harris et al., 2012). The three countries with the highest reported rates of CAM usage were all in East Asia: Malaysia, (76 %), Japan (76 %), South Korea (75 %) (Harris et al., 2012). Although an earlier study in 2004 by Barnes et al. among adult in the United States (US) revealed that 62 % of American adults may use some types of CAM, more recent surveys indicate that the prevalence is approximately 38 % (Barnes et al., 2004). In Europe, 52 % of Australians (Harris et al., 2012), 49 % of French, and 46 % of Germans reported using CAM therapy at some time in their life (Fjær et al., 2020, Kemppainen et al., 2018). In the Kingdom of Saudi Arabia (KSA), between 65 % and 80 % of people reported using CAM (Al-Faris et al., 2008, Elolemy and AlBedah, 2012), while the use of herbal medicine was 62.7 % (Syed et al., 2022). The most prevalent forms of herbal medicine are ginger extract (Al-Yousef et al., 2019, Syed et al., 2022 May), followed by garlic extract (Syed et al., 2022), cinnamon (Al-Yousef et al., 2019, Syed et al., 2022 May), and lemongrass (Syed et al., 2022).

Herbal medications may be beneficial, but concerns about quality and safety must be considered. The use of inferior raw materials with insufficient quality control measures may compromise the efficacy of herbal medicines. Herbal remedies are generally perceived as clean and natural, which makes them seem less risky than conventional medications. Unfortunately, it is not possible to conclude that these medications are totally devoid of problems such as toxicity and side effects. For example, several herbal remedies exacerbate the effects of anticoagulants and interact with cytochrome P450 enzyme systems, which are crucial to medication metabolism (Akeel et al., 2018, Al Akeel et al., 2018).

Safety is a key component of quality control and a vital element in the provision of herbal medicines and products for health care. The World Health Organization has published guidelines on how to safely use and monitor these medications (World Health Organization, 2004). Herbal medications are classified as non-prescription medicines or goods appropriate for self-care; therefore, prescriptions are not needed to purchase them in many countries. Typically, herbal medicine providers of CAM are not medical professionals. These include community pharmacists and nurses, as well as practitioners of CAM. Monitoring the safety of over-the-counter herbal medications should involve all providers of herbal medicines. The involvement of pharmacists and nurses in this field is growing, and they contribute significantly to safety monitoring. Medical professionals including doctors, nurses, and pharmacists might not be knowledgeable about the effects of herbal medicines on the health of their patients. Making informed decisions about diagnosis and treatment also requires an appropriate knowledge base. In addition, other healthcare professionals who do not routinely prescribe herbal remedies have limited knowledge about these products and how to utilize them. Herbal medicine education is also necessary for medical personnel who operate in poison control centers and health information services. Many consumers of CAM erroneously associate products from natural origins with the absence of adverse effects and subsequently do not consider it important to disclose their use of CAM to their physician. Similarly, individuals frequently neglect to disclose to their herbal medicine providers that they use other medications. In order to prevent potentially dangerous sequalae from the abuse of herbal medicines, education is essential for consumers, healthcare professionals, and herbal medicine providers.

Functional gastrointestinal disorders (FGID) are a class of conditions marked by persistent gastrointestinal (GI) symptoms, such as bloating, functional constipation, diarrhea, dyspepsia, and abdominal pain, even in the lack of obvious pathology on standard tests (Fikree and Byrne, 2021). GI symptoms are frequent, and the majority do not indicate a significant underlying illness (Stake-Nilsson et al., 2011). About 40 % of people globally have FGID, there is a higher prevalence in females, and it is less common in the elderly population (Fikree and Byrne, 2021). In Saudi Arabia, there is an estimated prevalence of dyspepsia, irritable bowel syndrome (IBS), constipation, Helicobacter pylori (H. pylori), and gastroesophageal reflux disease (GERD) of 92.4 %, 18 %, 43 %, 46.5 %, and 20 to 28.7 %, respectively (Akeel et al., 2018, Alhusainy et al., 2018, Alsuwat et al., 2018, Alwhaibi et al., 2020, Al Ghadeer et al., 2021, Alqahtani and Mahfouz, 2022).

Although FGID affects a significant portion of the general population, there are very few effective treatments. Treatment includes a biopsychosocial approach including diet and lifestyle modifications, treatment of associated psychiatric comorbidities, and medications to address underlying diseases (Fikree and Byrne, 2021). The majority of current therapies are prescribed to relieve symptoms rather than treating the underlying causes of the condition. Patients frequently seek alternative and complementary medical interventions because they are dissatisfied with traditional medical therapy (Chen et al., 2017). The use of alternative and complementary medicine varies based upon not only the type of disease, but also the culture and specific country of the patient (Chen et al., 2017). Its utilization in the field of gastroenterology is frequently seen among patients with FGID (Chen et al., 2017). The reported prevalence of CAM usage in London was 50.9 % for IBS and 49.5 % for inflammatory bowel disease (IBD) (Langmead, Chitnis and Rampton, 2002). The primary CAM therapies that have been used to treat FGID have involved dietary, drug/biological, psychological, herbal medicine, and behavioral therapies (Stake-Nilsson et al., 2011, Chen et al., 2017). Therefore, the aim of this study was to determine the prevalence of CAM utilization for FGID amongst the Saudi population and determine the types of CAM used for treatment.

## 2 Methods

### 2.1 Study design

A cross-sectional study was carried out in Riyadh, KSA during February 2023. Data was collected through a questionnaire published on social media platforms such as WhatsApp©️ and X©️ (formerly known as Twitter).

### 2.2 Sample size

Based upon the previous prevalence rate of CAM reported in KSA of 30 % (Elolemy. et al.,2012), a sample size of 323 was calculated using the following equation:

n = z^2^ × p × q/d^2^.

where n is the minimum sample size, z is the constant (1.96), p is the prevalence of CAM among the Saudi population (30 %), q is (1 − p), Z is the standard normal deviation of 1.96 corresponding to the 95 % confidence interval, and d is the desired degree of accuracy or tolerated margin of error which is 5 % (0.05).

### 2.3 Questionnaire development

Data was gathered using a structured self-questionnaire prepared in Arabic. The questionnaire used in this study was created after thoroughly evaluating the literature on FGID, CAM use, and types of CAM using for FGID. There were three sections in the questionnaire: (1) demographic information, (2) questions to determine the prevalence of CAM use within the past month and current use of FGID, the types of FGID, and the types of CAM, and (3) questions on the sources of information about CAM. The first draft of the questionnaire was reviewed by research experts in the pharmacy practice field. Then, a pilot study was conducted in a randomly selected sample of 26 respondents to get their feedback on how to make the questionnaire more user-friendly. The Cronbach’s alpha was 0.731. The data was collected using convenience sampling by using social media as the main platform for data collection. For the purpose of data collection, a researcher was appointed. The data was collected until the required number of responses obtained by sending reminders for completing the questionnaires. In addition to obtaining the maximum number of responses, a snowball technique was utilized, where a participant who received the survey would suggest or recruit participants.

### 2.4 Data analysis

The data was analyzed using the Statistical Package for the Social Sciences (SPSS) (version 26) for Windows (SPSS Inc., Chicago, IL, USA). Descriptive statistics were used to summarize the demographic features. The Chi-square test was employed to investigate the relationship between the demographic data and CAM use. Bivariable and multivariable logistic regressions were applied to determine factors associated with CAM use.

## 3 Results

A total of 828 people participated in this study. Of the 828 participants, 601 (72.6 %) were males. The majority of respondents reported that their age was between 18 to 25 years old. Approximately 42 % of respondents held a university degree. Table 1 provides more details on the respondents’ demographic data. The overall prevalence of CAM use for FGID problems was 87.2 %. There was no significant difference in CAM use for FGID problems between men (87.5 %) and women (86.3 %) (P = 0.727). Among age groups, adults in the age group greater than 55 years used CAM significantly more (97.3 %) than other age groups (P < 0.001).

**Table 1:** Univariable and bivariable analyses of CAM utilization for FGID among Saudi participants (N = 828).

Demographic data	Total n = 828 n (%)	CAM use n = 722 (87.2 %) n (%)	No CAM use n = 106 (12.8 %) n (%)	*P* value
**Gender** Male Female	601 (72.6) 227 (27.4)	526 (87.5) 196 (86.3)	75 (13.7) 31 (13.7)	0.727
**Age (years)** <18 years 18–25 26–35 36–45 46–54 > 55	6 (0.70) 321 (38.8) 201 (24.3) 106 (12.8) 81 (9.8) 113 (13.6)	1 (16.7) 258 (80.4) 176 (87.6) 101 (95.3) 76 (93.8) 110 (97.3)	5 (83.3) 63 (19.6) 25 (12.4) 5 (4.7) 5 (6.2) 3 (2.7)	<0.001
**Education** Illiterate Intermediate High school University Postgraduate	73 (8.8) 110 (13.3) 168 (20.3) 348 (42.0) 129 (15.6)	73 (1 0 0) 83 (75.5) 147 (87.5) 294 (84.5) 125 (96.9)	0 27 (24.5) 21 (12.5) 54 (15.5) 4 (3.1)	<0.001
**Employment status** Students Government Employed Unemployed Retired Private employer	283 (34.2) 301 (36.4) 66 (8.0) 105 (12.7) 73 (8.8)	253 (89.4) 233 (77.4) 59 (89.4) 104 (99.0) 73 (1 0 0)	30 (10.6) 68 (22.6) 7 (10.6) 1 (1.0) 0	<0.001
**Insurances** Governmental hospital Private Both None	326 (39.4) 206 (24.9) 67 (8.1) 229 (27.7)	283 (86.8) 178 (86.4) 65 (97.0) 196 (85.6)	43 (13.2) 28 (13.6) 2 (3.0) 33 14.4)	0.09

**Fig. 1:**
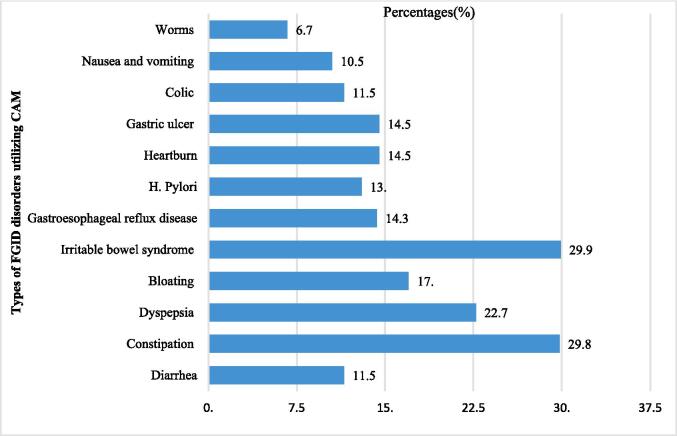
Types of FGID disorders utilizing CAM among Saudi participants (n = 722).

The most common FGID disorders for utilizing CAM were IBS (29.9 %), followed by constipation (29.8 %), dyspepsia (22.7 %), bloating (17.0 %), heartburn (14.5 %), gastric ulcer (14.5 %), H. pylori (13.0 %), colic (11.5 %) and nausea/vomiting (10.5 %) as shown in Fig. 1.

**Fig. 2:**
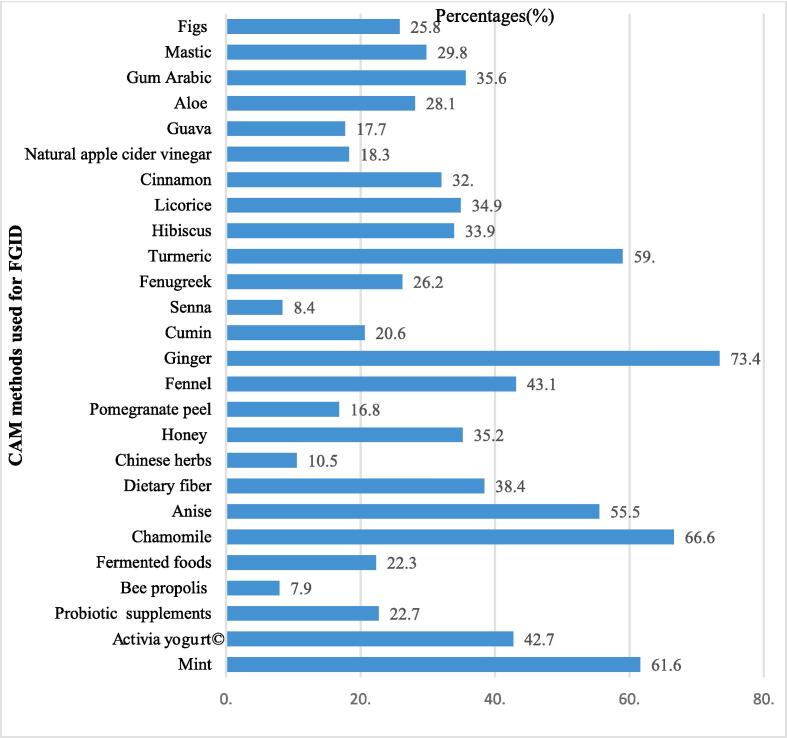
CAM methods used for FGID (n = 722).

The most commonly used types of CAM for FGID were ginger (73.4 %), chamomile (66.6 %), mint (61.6 %), turmeric (59.0 %), anise (55.5 %), fennel (43.1 %), Activia yogurt©️ (42.7 %), dietary fiber (38.4 %), honey (35.2 %), licorice (34.9 %), hibiscus (33.9 %), and cinnamon (32.0 %). Fig. 2 displays the most common types of CAM for FGID.

Table 2 shows the bivariable logistic regression of factors associated with the utilization of CAM. In the bivariable regression, gender did not have an impact on the odds of using CAM. Subjects aged < 25 years had a significant odds ratio of CAM use compared to participants aged 26–35 (OR = 1.85; 95 % CI: 1.12–3.04), 36–45 (OR = 5.30; 95 % CI: 2.08–15.53), 46–55 (OR = 3.99; 95 % CI: 1.55–10.25) and > 55 years (OR = 9.63; 95 % CI: 2.97–31.25). Compared to an educational level of less than high school, participants who completed postgraduate education were significantly more likely to use CAM (OR = 5.41; 95 % CI: 1.84–15.86). When compared to students, employed and retired participants had a significant odds ratio of using CAM (OR = 0.41; 95 % CI: 0.26–0.65) and (OR = 12.33; 95 % CI: 1.66–91.62), respectively. Participants who had both public & private insurances had significant odds ratios (OR = 5.47; 95 % CI: 1.28–23.43) compared to those with only one type of insurance.

**Table 2:** Bivariable and multivariate logistic regression of factors associated with utilization of CAM.

Characteristics	OR	95 % CI		P value
		Lower	Upper	
Male	1.11	0.71	1.74	0.651

**Age**				
<25 y**	1.00			
26–35 y	1.85	1.12	3.04	0.015*
36–45 y	5.30	2.08	13.53	<0.001*
46–54 y	3.99	1.55	10.25	0.004*
>55 y	9.63	2.97	31.25	<0.001*

**Educational level**				
Less than high school**	1.00			
High school	1.21	0.66	2.24	0.540
University	0.94	0.57	1.56	0.816
Postgraduate	5.41	1.84	15.86	0.002*
Employment status				
Students**	1.00			
**Employer**	0.41	0.26	0.65	<0.001*
Unemployed	1.00	0.42	2.39	0.999
Retired	12.33	1.66	91.62	0.014*
Insurance				
None**	1.00			
Public	1.11	0.68	1.81	0.681
Private	1.07	0.62	1.84	0.806
Both	5.47	1.28	23.43	0.022*
Multivariable logistic regression of factors associated with utilization of CAM				

**Age**				
<25 y**	1.00			
26–35 y	0.82	0.27	2.57	0.739
36–45 y	1.05	0.23	4.80	0.952
46–54 y	0.58	0.12	2.82	0.501

**Educational level**				
Less than high school**	1.00			
High school	2.73	1.22	6.13	0.015*
University	4.18	2.03	8.58	<0.001*
Postgraduate	20.85	5.51	78.80	<0.001*

**Employment status**				
Students**	1.00			
Employer	0.84	0.23	3.05	0.793
Unemployed	1.66	0.48	5.74	0.425
Retired				

**Insurance**				
None**	1.00			
Public	0.71	0.39	1.30	0.273
Private	0.27	0.14	0.55	<0.001*
Both	3.43	0.68	17.27	0.134
* Significant p value				
** Used as a reference				

Table 2 shows the multivariable logistic regression of factors associated with utilization of CAM. In the bivariable regression, age and employment status did not have an impact on the odds of using CAM. The subjects who had high school, university, and postgraduate education had significant odds ratios of CAM use (OR = 2.73; 95 % CI: 1.22–6.13), (OR = 4.18; 95 % CI: 2.03–8.58), and (OR = 20.85; 95 % CI: 5.51–78.80), respectively, compared to subjects who did not complete high school. Participants who had private insurance had a significant odds ratio (OR = 0.27; 95 % CI: 0.14–0.55) compared to governmental insurance; however, Hosmer–Lemeshow test was not significant (=0.370).

**Fig. 3:**
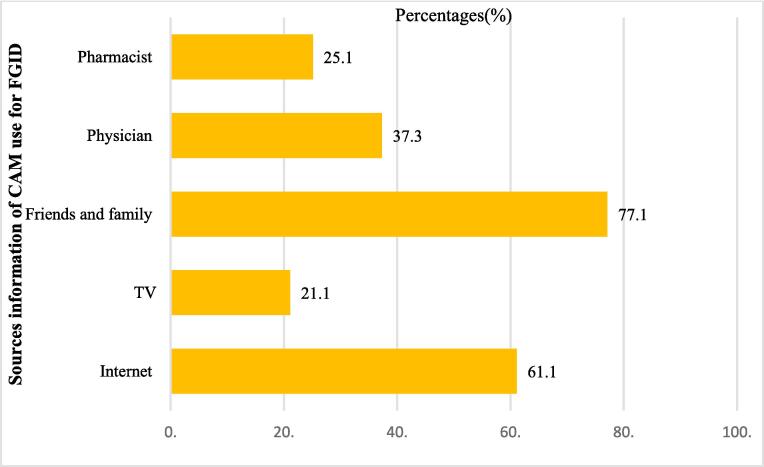
Sources information of CAM use for FGID.

The major sources of CAM information for FGID were friends/family (77.1 %), the internet (61.1 %), and pharmacists (25.1 %) (Fig. 3).

## 4 Discussion

According to our knowledge, this is the first study conducted to determine the prevalence of use of CAM to treat gastrointestinal disorders in KSA. Previous studies have focused primarily on the prevalence of the use of CAM in KSA. A recent national survey was conducted by Al-Jawadi et al. among elderly people to determine the prevalence of the use of alternative medicine. This study found that the prevalence of CAM use was 62.5 % (Aljawadi et al., 2020). Another study was conducted in Riyadh, KSA among 740 adults to determine gender differences in CAM utilization according to personal practices and opinions. It was reported that approximately 36.0 % of participants used CAM (Abdalla et al., 2020).

In this study, the prevalence of CAM use for GI disorders was high amongst the Saudi population (87.2 %). Similar to the differences in languages, traditional medications, and cultures, the prevalence of CAM use for GI disorders differs between studies and countries. For instance, the prevalence of CAM use for GI disorders among adults has been reported to be between 23 % to 44 % and 63.5 % for American and Australian adults, respectively (Gan et al., 2018). Yoon et al. utilized an online survey to identify the self-reported use of CAM, including dietary supplements, to treat GI symptoms and found that 84.5 % of US respondents used herbal medicines (Yoon et al., 2018).

The multivariable logistic regression showed an excellent fit, according to the Hosmer-Lemeshow test (P = 0.370). Regression analysis showed that there were no noticeable differences in CAM use by gender. This result is similar to several published studies (Thomas and Coleman, 2004, Jawahar et al., 2012, Abdalla et al., 2020, Aljawadi et al., 2020). Nonetheless, a number of studies have reported that adolescent girls are more likely to use CAM (Kristoffersen et al., 2014, Alwhaibi and Sambamoorthi, 2016, Carlsson et al., 2020). This study showed that CAM utilization may be influenced by a combination of factors including education levels, income, and other socioeconomic categories like employment status. Previous studies have reported that the use of CAM was more common among people with higher educational levels (Grundmann, 2014). Contrary to that, a study conducted in KSA revealed that participants with low levels of education were more likely to use CAM than those with higher education levels (Syed Faisal Zaidi et al., 2022).

In the present study, the most common type of CAM used for FGID was ginger (73.4 %). Ginger is a valued dietary supplement that has carminative action, decreases intestinal pains, lowers the strain on the lower esophageal sphincter, and guards against bloating, gas, and dyspepsia (Nikkhah Bodagh, Maleki and Hekmatdoost, 2018). It has been reported that the most common CAM utilized for IBS is ginger (van Tilburg et al., 2008). About 62 % of participants used peppermint extracts for FGID. It has been reported that peppermint leaves are commonly used to treat digestive disorders symptomatically (Begas et al., 2017). In addition, peppermint is one of the most extensively studied natural CAM remedies for the treatment of functional dyspepsia, bloating, and epigastric pain, and has many effects such as antiemetic properties and a spasmolytic effect (Begas et al., 2017, Deutsch et al., 2020). It was interesting that most of our subjects revealed that they use turmeric for FGID. According to a *meta*-analysis that involved five clinical trials, curcumin, turmeric’s major active ingredient, was shown to be generally effective in decreasing IBS symptoms (Lopresti et al., 2021). Also, curcumin can be used as an adjuvant in the treatment of H. pylori infection (Kwiecien et al., 2019). Approximately 43 % of subjects consumed probiotic yogurt (Activia yogurt©️) for FGID. Studies have reported that probiotic yogurt is effective for gut health, including disorders such as FGID, IBS, and ulcerative colitis, and patients reported that it significantly reduced their stomach discomfort and flatulence (Kim et al., 2019, Hadjimbei et al., 2022). About 35 % of subjects reported that they used honey to treat FGID. This finding is supported by the clinical literature. Honey has been proposed as a possible treatment for a number of gastrointestinal tract diseases including dyspepsia, periodontal disease, and other oral problems. It has also been considered as an effective component of oral rehydration therapy. According to in vitro research, honey may have bactericidal effects on H. pylori (Samarghandian et al., 2017, Schell et al., 2022). A summary of evidence-based recommendations related to selected CAM therapies can be found in Table 3.

**Table 3:** An evidence-based summary of selected CAM methods.

**CAM methods**	Uses	Contraindications	Interactions	Adverse reactions	Dosing
**Mint**	GI conditions including nonferrous constipation or diarrhea associated with Irritable bowel syndrome to reduce global symptoms of pain and bloating	Patients with gastroesophageal reflux or active gastric ulcers due to decreased esophageal sphincter pressure	May inhibit CYP-450 3A4	May worsen symptoms of heartburn, hiatus hernias, and stomach ulcers	Nonserious constipation and diarrhea associated with IBS: Up to 1200 mg/day (capsules containing 180 to 200 mg − 1 to 2 capsules 3 times daily) for 2 to 4 weeks; IBS symptoms: 180 mg 3 times daily for 4 weeks; Studies have used capsule formulations recommended to be taken 30 to 60 min before meals on an empty stomach
**Activia yogurt©️ and probiotic supplements**	Irritable bowel syndrome	Severe acute pancreatitis; not recommended in patients at risk for opportunistic infections and those with badly damaged GI tracts	None well documented	Relatively safe; abdominal cramping, nausea, fever, soft stools, flatulence, and taste disturbances have been reported	IBS: Up to 5 billion units daily taken for 4 to 6 weeks; The majority of trials used daily dosing; however, twice weekly dosing has been used
**Bee propolis**	GI disturbances	Not recommended in children younger than 12 months due to the potential for botulism	None well documented	Allergic reactions to pollen may occur when honey is ingested. Some case reports of acute hepatitis following ingestion of bee pollen have been reported. Case reports exist of allergy, acute exacerbation of asthma, anaphylaxis, and death. Case reports of propolis-induced fixed-drug eruption have been published.	The type of product, plant species of origin, methods of processing, and dose form have all been shown to affect the appropriate dose and duration, safety, and effectiveness
**Chamomile**	Nausea and vomiting of pregnancy; ulcerative colitis	Hypersensitivity to ragweed pollens	May enhance the anticoagulant effect of warfarin	Anaphylaxis, dermatitis, and other hypersensitivity reactions; cross reactivity among people with allergies to ragweed, asters, chrysanthemums, and other members of the Asteraceae family.	Clinical data are lacking to support specific dosing recommendations
**Anise**	GI effects (bloating, discomfort, pain, diarrhea, constipation)	Not recommended for use in pregnancy in amounts exceeding those found in food.	None well documented	May cause allergic reactions of the skin, respiratory tract, and GI tract.	Clinical data are lacking to support specific dosing recommendations
**Honey**	Diarrhea; peptic ulcers	Not recommended in children younger than 12 months due to the potential for botulism	None well documented	Allergic reactions to pollen may occur when honey is ingested. Some case reports of acute hepatitis following ingestion of bee pollen have been reported. Case reports exist of allergy, acute exacerbation of asthma, anaphylaxis, and death. Case reports of propolis-induced fixed-drug eruption have been published.	The type of product, plant species of origin, methods of processing, and dose form have all been shown to affect the appropriate dose and duration, safety, and effectiveness
**Pomegranate peel**	Diarrhea	Not identified	Avoid in combination with bosutinub, panobinostat, ribociclib, and sildenafil	Fecal impaction as a result of pomegranate seed bezoars has been reported; 3 cases of priapism have occurred subsequent to concomitant use of sildenafil 50 mg and pomegranate juice	N/A
**Fennel**	Used as stimulant and carminative agent	Not identified	None well documented	Fennel may cause photodermatitis, contact dermatitis, and cross reactions. The oil may induce reactions, such as hallucinations and seizures. Four cases of premature thelarche (breast development) in girls have been reported with the use of fennel.	Fennel seed and fennel seed oil have been used as stimulant and carminative agents in doses of 5 to 7 g and 0.1 to 0.6 mL, respectively.
**Ginger**	Prevention and management of nausea; irritable bowel syndrome	Not identified	Anticoagulants (eg, warfarin), agents with antiplatelet properties, nonsteroidal anti-inflammatory drugs (NSAIDs), salicylates, thrombolytic agents, antihypertensives, hypoglycemic agents, and crizotinib interact with ginger.	Large doses carry the potential for adverse reactions. Mild GI effects (eg, heartburn, diarrhea, mouth irritation) have been reported, and case reports of arrhythmia and immunoglobulin E (IgE) allergic reactions have been documented.	Nausea and vomiting: Various doses and durations; irritable bowel syndrome: 1 to 2 g daily for 28 days
**Cumin**	Diarrhea; colic; flatulence	Not identified	None well documented	May cause hypoglycemia	N/A
**Senna**	Stimulant laxative	Senna is contraindicated in patients with intestinal obstruction, ulcerative colitis, appendicitis, and Crohn disease. Senna is not recommended for children younger than 2 years.	Avoid use of senna with drugs known to deplete potassium.	Senna may cause diarrhea, loss of fluids, hypokalemia, and abdominal pain/cramping. The long-term use of senna has resulted in pigmentation of the colon, reversible finger clubbing, cachexia, and laxative dependence. Children, particularly those wearing diapers, may experience severe diaper rash, blister formation, and skin sloughing	Senna leaves and pods have been used as a stimulant laxative at dosages of 0.6 to 2 g/day, with a daily dose of sennoside B from 20 to 30 mg. A bitter tea can be made containing senna 0.5 to 2 g (0.5 to 1 teaspoon).
**Fenugreek**	Heartburn	Contraindications have not been identified. Avoid use in individuals with allergy to any member of the Fabaceae family. Cross-reactivity to chickpea, peanut, or coriander allergy is possible.	Interactions with hypoglycemia-associated agents, sertraline, and warfarin are possible; monitor therapy.	Mild and transient GI effects are most commonly reported; hypoglycemia, micturition, and dizziness have also been documented within a range of doses and variety of preparations. Allergy to fenugreek is recognized and includes severe responses such as asthma, anaphylaxis, and toxic epidermal necrolysis. Cross-reactivity to legumes is possible; consider allergy potential with chickpeas, peanuts, soybeans, lentils, lupin, green peas, or coriander. Fenugreek has generally recognized as safe (GRAS) status as a food additive (eg, when used as a spice in cooking). When ingested in culinary quantities, fenugreek is usually devoid of adverse reactions.	N/A
**Turmeric**	irritable bowel syndrome ulcerative colitis dyspepsia	Use is contraindicated if hypersensitive to any of the components of curcumin. Avoid use during pregnancy and lactation because of emmenagogue and uterine stimulant effects. Turmeric should not be used in patients with gallstones or bile duct or passage obstruction.	Potentially interacts with CYP2D6 and CYP3A substrates, antiplatelet agents, anticoagulants, cladribine, nonsteroidal anti-inflammatory agents, salicylates, and thrombolytic agents.	Clinical trials report few adverse reactions (eg, dyspepsia, pruritus). Rare cases of contact dermatitis and anaphylaxis have also been reported.	Irritable bowel syndrome:1,800 or 3,600 mg/day Crohn disease: 3 g/day plus azathioprine for 6 months Ulcerative colitis: curcumin (1 g twice daily in combination with standard mesalamine or sulfasalazine therapy) for maintenance of remission; add-on curcumin (95 % pure, 1.5 g twice daily) for induction of remission for 1 month; gastric pain, dyspepsia symptoms, and confirmed gastric or duodenal ulcers in the presence of Helicobacter pylori: In addition to standard H. pylori triple therapy, patients received curcumin 500 mg plus piperine 5 mg daily; Functional dyspepsia and no previous history of infection with gastric H. pylori: curcumin (250 mg) alone, omeprazole (20 mg) alone, and the curcumin/omeprazole combination groups for 28 and up to 56 days; functional dyspepsia: 750 to 1,500 mg/day (in divided doses 3 times daily 30 to 60 min after each meal of the day) for 4-weeks
**Hibiscus**	Mild laxative	Not identified	Studies in healthy volunteers have shown altered chloroquine, acetaminophen, and diclofenac pharmacokinetics. The clinical effects of these interactions have not been evaluated. Interactions are also possible with blood pressure–lowering agents, caffeine and caffeine-containing products, diclofenac (systemic), erlotinib, herbs with hypotensive properties, and simvastatin.	Well tolerated	N/A
**Licorice**	H. pylori positive patients with peptic ulcer disease; gastric irritation	Not identified	Atogepant Antihypertensive agents Carbamazepine ClozapineCorticosteroids (systemic) Hormonal contraceptives Loop diuretics Methotrexate Midazolam Nimodipine Selpercatinib Sirolimus SolriamfetolTacrolimus (systemic) Thiazide and thiazide-like diuretics Ubrogepant TriazolamYi-gan san	At lower dosages or normal consumption levels, few adverse reactions are evident. Ocular effects and hypersensitivity have been described. Hypertension and hypokalemia are recognized effects of excessive licorice consumption.	Licorice root extract as a 380 mg tablet administered twice daily for 2 weeks was evaluated in patients who were H. pylori positive with peptic ulcer disease.
**Cinnamon**	Diarrhea, dyspepsia, irritable bowel syndrome	Use is contraindicated in individuals allergic to cinnamon	None well documented	Allergic reactions after heavy exposure	N/A
**Natural apple cider vinegar**	Dyspepsia; gastroparesis	Severe allergies to apples	None documented	Primarily related to topical applications	Not well established
**Guava**	Diarrhea	Specific contraindications have not been identified; however, hypersensitivity should be considered a contraindication.	None well documented	No serious adverse reactions have been reported in limited clinical trials.	500 mg every 8 h for 3 days
**Aloe**	Gastrointestinal/Inflammatory bowel disease	contraindicated in women who are pregnant or breast-feeding, children younger than 12 years of age, and elderly patients with suspected intestinal obstruction	According to a statement from the American Heart Association, aloe taken orally may reduce potassium levels and increase the risk of digoxin toxicity.Hypoglycemic-associated agents: Herbs (hypoglycemic properties) may enhance the hypoglycemic effect of hypoglycemic-associated agents. Monitor therapy.	A case of small bowel obstruction from aloe bezoars, as well as acute hepatitis induced by aloe vera ingestion, has been reported.	Various doses; 250 mg twice daily for 4 week has been used
**Gum Arabic**	Various GI conditions (chronic diarrhea, GI protection for NSAID use, fecal incontinence)	Not identified	None well documented	Allergic reactions have been reported. Adverse effects reported in clinical trials included unfavorable sensation in the mouth, early morning nausea, mild diarrhea, and bloating.	Several trials used gum arabic 30 g orally daily for 6 to 12 weeks for various indications.
**Mastic**	Irritable bowel disease	Individuals with hypersensitivity to pollen or to any of the ingredients of mastic gum.	None well documented	Related to hypersensitivity to the plant species or allergic reactions	14 mg to 0.37 g have been used for 4 weeks to 3 months

Adapted from UpToDate Lexi-Drugs, 2024® and NatMedPro, 2024®.

Physicians and pharmacists were identified as trusted sources of information regarding CAM use. Although physicians are often consulted initially, approximately only 25 % receive education about CAM in required medical college courses and only 15 % were exposed to CAM during residency training (Patel, Kemper et al. 2017). Furthermore, post-graduate physician residents reported a “don’t ask, don’t tell” culture between patients and their physicians. Although they believed that up to 40 % of patients use CAM, they believed that less than 20 % spontaneously divulge CAM use and they estimate that hey routinely ask patients about CAM use less than 20 % of the time. Physician interest in learning about CAM also tends to decline after the preclinical years of medical school (Joyce, Wardle et al. 2016). Foley et al. conducted a systematic review and *meta*-analysis of the rate of disclosure of CAM use to medical providers (i.e. primarily physicians) and reported that 67 % of patients do not inform their physicians about CAM use (Foley, Steel et al. 2019).

Pharmacists are considered one of the most important healthcare providers well-suited to give advice to the users of CAM by presenting evidence-based medical counseling to ensure the safety of these products (Farrell, Ries et al. 2008). Pharmacists are well positioned to inform patients on the usage, effectiveness, side effects, and possible interactions with prescription drugs with dietary and herbal supplements regardless of how these products are regulated (Ng et al., 2021). Moreover, the majority of dietary and herbal supplements users are more likely to confide in pharmacists about their use of these products than to their physicians (Kheir, Gad et al. 2014). A study carried out in Australia found that 87–92 % of customers thought pharmacists could give them sufficient, trustworthy information on the efficacy and safety of dietary and herbal supplements (Mehralian, Yousefi et al. 2014). It must be emphasized that it is important for pharmacists to have adequate knowledge regarding these products in order to provide patients with accurate recommendations about dietary and herbal supplements (Ng, Tahir et al. 2021).

The authors would like to acknowledge some limitations of the study. This was a cross-sectional study and the possibility of selection biases cannot be ruled out. Participation of specific populations such as the elderly and patients of lower socioeconomic statuses may have been limited due to lack of access to the survey. The possibility of recall bias may have influenced the results since self-reported data was used. Furthermore, there may have been demographical bias due to the fact that FGID is most commonly reported in females; however, a larger proportion on males reported symptoms in our study. Therefore, these limitations may affect the generalizability of study findings.

## 5 Conclusion

The use of CAM among the Saudi population is alarmingly high; however, the lack of standardized medical recommendations and treatment options may be the cause. Although there were no significant gender differences, participants with higher educational levels and private insurance coverage were more likely to use CAM for FGID. Patients suffering from FGID and limited access to medical advice and treatment options are vulnerable to being exposed to dubious and incredible information sources. Expanding access to preventative medical services, funding governmental medical websites to provide credible information, educating healthcare professionals about FGID, and conducting more research in safe and effective treatments for FGID is recommended.

## CRediT authorship contribution statement

**Salmeen D. Babelghaith:** Conceptualization, Data curation, Formal analysis, Methodology, Project administration, Writing – original draft. **Ibrahim Sales:** Writing – review & editing. **Wajid Syed:** Formal analysis, Writing – review & editing. **Mohamed N. Al-Arifi:** Conceptualization, Methodology, Supervision, Visualization, Writing – review & editing.

## Declaration of Competing Interest

The authors declare that they have no known competing financial interests or personal relationships that could have appeared to influence the work reported in this paper.
